# Calibrated microphone array recordings reveal that a gleaning bat emits low-intensity echolocation calls even in open-space habitat

**DOI:** 10.1242/jeb.245801

**Published:** 2023-09-27

**Authors:** Léna de Framond, Thejasvi Beleyur, Daniel Lewanzik, Holger R. Goerlitz

**Affiliations:** ^1^ Acoustic and Functional Ecology, Max Planck Institute for Ornithology, 82319 Seewiesen, Germany; ^2^ Department of Biology, University of Konstanz, 78464 Konstanz, Germany; ^3^ Centre for the Advanced Study of Collective Behaviour, University of Konstanz, 78464 Konstanz, Germany

**Keywords:** Brown long-eared bats, Source level, Evolutionary arms race, *Plecotus*, Gleaning, Call amplitude

## Abstract

Echolocating bats use ultrasound for orientation and prey capture in darkness. Ultrasound is strongly attenuated in air. Consequently, aerial-hawking bats generally emit very intense echolocation calls to maximize detection range. However, call levels vary more than tenfold (>20 dB) between species and are tightly linked to the foraging strategy. The brown long-eared bat (*Plecotus auritus*) is a primarily gleaning, low-amplitude species that may occasionally hawk airborne prey. We used state-of-the-art calibrated acoustic 3D-localization and automated call analysis to measure *P. auritus*’ source levels. *Plecotus auritus* emits echolocation calls of low amplitude (92 dB rmsSPL re. 20 µPa at 10 cm) even while flying in open-space. While *P. auritus* thus probably benefits from delayed evasive manoeuvres of eared insects, we propose that low-amplitude echolocation did not evolve as an adaptive countermeasure, but is limited by morphological constraints.

## Teaser

**Summary:** Brown long-eared bats in free flight emit low echolocation calls (92 dB rmsSPL re. 20 µPa at 10 cm) even in open space, which severely constraints their sensory range.

## INTRODUCTION

The information that an animal can obtain about its environment is limited by the maximum detection range over which it can detect sensory stimuli. This detection range is determined by properties of the environment, the stimulus and the sensory system (Dusenbery, 2001). The ‘sensory drive hypothesis’ postulates that both signals and sensory systems are adapted to an animal's typical environment to maximize information acquisition (Cummings and Endler, 2018; Endler, 1992).

Echolocating bats perceive their surroundings through sound. Open-space foraging species maximize detection range by emitting very high-intensity calls with source levels often above the human pain threshold [120–140 dB peak-equivalent sound pressure level (peSPL) re. 20 µPa at 10 cm distance to the mouth; Boonman et al., 2013; Fujioka et al., 2014; Goerlitz et al., 2020; Holderied and von Helversen, 2003; Holderied et al., 2005; Surlykke and Kalko, 2008]. Morphological and behavioural adaptations, such as large ears (Coles et al., 1989; Wotton et al., 1995) and ear movements (Yin and Müller, 2019), aid in prey detection. Nevertheless, their detection range is limited to between a few metres to tens of metres (Stilz and Schnitzler, 2012), strongly depending on call frequency, amplitude, object size and reflective properties (Møhl, 1988).

In contrast to open-space foraging bats, some species catch prey from vegetation or the ground (‘gleaning’) and typically emit low-amplitude calls, more than 10 times (>20 dB) fainter than those of aerial-hawking species (Corcoran and Conner, 2017; Goerlitz et al., 2010; Holderied et al., 2011; Seibert et al., 2015; Stidsholt et al., 2022). Using low-amplitude echolocation during gleaning avoids masking of the faint prey-generated sounds and prey echoes by loud vegetation echoes and is regarded as an adaptation to foraging close to background structures (Jakobsen et al., 2013; Surlykke et al., 2009). For example, the desert long-eared bat (*Otonycteris hemprichii*, plecotine tribe) emits low-amplitude echolocation calls during ground gleaning but increases call amplitude drastically when flying in the open (Hackett et al., 2014), suggesting that the shorter detection distance of low-amplitude calls is costly during aerial hawking. In contrast, the Western barbastelle bat (*Barbastella barbastellus*) and the North-American Townsend's big-eared bat (*Corynorhinus townsendii*) emit low-amplitude calls (∼100 dB peSPL at 10 cm) in open space (Corcoran and Conner, 2017; Lewanzik and Goerlitz, 2018, 2021). Although this low-amplitude strategy strongly limits these bats' detection distance for prey, it provides an advantage over eared moths, which fail to hear their predator and to initiate evasive flight (Corcoran and Conner, 2017; Goerlitz et al., 2010; Lewanzik and Goerlitz, 2018).

Brown long-eared bats (*Plecotus auritus*), like most other plecotine species, glean prey off vegetation and emit low-amplitude calls when gleaning (Anderson and Racey, 1991; Waters and Jones, 1995). The high proportion of eared-moths in their diet (∼25–40%; Rydell, 1989; Shiel et al., 1991; Vesterinen et al., 2018) and observations of hawking in the lab (Anderson and Racey, 1991) suggest that *P. auritus* might also hawk prey in open space (Dietz and Kiefer, 2016). To date, it is unknown whether they emit low-intensity calls when flying in open space – like the barbastelle and Townsend's big-eared bat – or high-intensity calls – like desert long-eared bats.

To close this gap of knowledge, we measured call source levels of wild *P. auritus* flying freely in their natural open-space habitat. We show that *P. auritus* emit low-intensity calls in open space, and discuss hypotheses about the evolutionary drivers underlying low-intensity echolocation.

## MATERIALS AND METHODS

### Field recordings

We caught 10 (8 males, 2 females) brown long-eared bats, *Plecotus auritus* (Linnaeus 1758), with mist-nets on 21 August 2019, next to a large bat colony at Silberberg, Eastern Bavaria, Germany. Bats were briefly kept in cloth bags. Within ∼10–15 min, we released bats individually from the hand at ∼10 m distance to a microphone array. Five of the bats flew towards the microphone array, allowing us to record their echolocation call sequences, which we used for subsequent reconstruction of their spatial positions and call analysis. The array consisted of four microphones (FG-O, Avisoft Bioacoustics, Glienicke, Germany) arranged in a planar symmetrical star-shaped pattern, with one central microphone surrounded by three microphones at 60 cm distance and 120 deg angular separation. The microphone array was positioned on the top of a small hill, free of trees, so that the bats were flying in open surroundings. Audio was recorded via a USG-416H soundcard and Recorder software (Avisoft Bioacoustics) at 500 kHz sampling rate and 16-bit resolution. Ambient temperature, relative humidity and atmospheric pressure were logged every 2 min during the recording session (Kestrel 4000, Nielsen-Kellerman, Boothwyn, PA, USA).

### Flight path reconstruction and call analysis

We used custom-written software (TOADSuite, by Peter Stilz; Hügel et al., 2017; Lewanzik et al., 2019) for MATLAB 2007b (The MathWorks Inc., Natick, MA, USA) to calculate the bat's 3D position for each emitted call, and to analyse each call's acoustic properties as emitted by the bat. First, all recordings were filtered (20–90 kHz fourth-order elliptic bandpass filter, 0.1 dB peak-to-peak ripple, 40 dB minimum stopband attenuation) and calls were detected on the central microphone above a fixed threshold of −35 dB full scale (FS, i.e. relative to the maximum recordable level). Second, for each call, we measured the time-of-arrival difference between the central and each peripheral microphone by cross-correlation, and calculated the bat's 3D position at call emission. We displayed all detected bat positions (*N*=144) and their corresponding call waveforms and spectrograms on a graphical user interface to manually combine positions into flight trajectories and to visually control data quality. We manually excluded outlier positions (that did not align well with or were far off the other positions, *N*=16), and automatically excluded all positions of calls with a duration <1 ms (i.e. shorter than typical species-specific echolocation calls) or >8 ms (i.e. twice as long as typical echolocation calls; Russ, 2012). The >8 ms calls were measurement errors due to low signal to noise ratio (<15 dB, we manually checked all these calls; see Supplementary Materials and Methods; *N*=30 excluded calls). Finally, 98 calls remained for the final analysis.

To analyse the acoustic call parameters as emitted by the bat (at 10 cm distance from its mouth), we first reconstructed the emitted waveform of each detected call by correcting for microphone characteristics and sound attenuation on the way to the microphone. The microphone's frequency response and sensitivity were previously measured by recording white noise and pure tones in comparison to a calibrated measuring microphone (for details see Supplementary Materials and Methods). We calculated each call's recorded amplitude and phase spectrum with a fast Fourier transformation (FFT), corrected the amplitude spectrum for the microphone's frequency response, for frequency- and distance-dependent atmospheric attenuation at the local weather conditions (Goerlitz, 2018), and for the distance-dependent geometric attenuation, and then back-calculated the compensated waveform with an inverse FFT. We used the reconstructed source waveform to calculate the call's duration (at −12 dB below the peak of the call's envelope smoothed with a moving average of 0.2 ms) and apparent source level (aSL). For better comparability with other studies, we calculated the apparent source level both as peSPL (i.e. a measure of the call's peak-to-peak amplitude; Burkard, 2006) and as root-mean-square sound pressure level (rmsSPL, i.e. a measure of the call's average amplitude). The peSPL value was calculated from the envelope of the call waveform, and the rmsSPL value as the root-mean-square of the waveform within the −12 dB call duration criterion. All sound pressure levels are referenced to 20 µPa and 10 cm distance to the bat's mouth. The aSL underestimates the real or ‘on-axis’ source level as the bats' flight and sonar beam direction are not always oriented towards the microphone. To estimate the real, on-axis source level, we calculated the 95th percentile of all aSL values per trajectory. This method is less likely to be influenced by outliers than calculating the mean of the 10% most intense calls or taking the maximum value per trajectory. A single very large value would not change the 95th percentile but would have a substantial impact on the mean or maximum (Holderied and von Helversen, 2003; Surlykke and Kalko, 2008). We further calculated each call's peak frequency (frequency with maximum amplitude of the average spectrum) and lowest frequency (lowest frequency at −12 dB below the peak frequency's amplitude) from the time-averaged call spectrogram (2000 FFT of 100 samples with Hann window, 95% overlap) derived from the compensated call waveform. To check whether the bats altered their echolocation to the presence of the microphone array, we fitted a linear mixed effect model (package *rstanarm* version 2.21.3, R version 4.2.2) on call duration over distance. We checked for the typical call duration decrease during object approach, limiting the analysis to calls within 3 m of the array, where we expected the strongest change in call parameters. The model was fitted in a Bayesian framework (4 chains of 5000 iterations, warmup 2500 iterations, default uninformative flat priors) with log(call duration) as response variable, distance to the central microphone as fixed effect, and bat identity as a random effect. We checked that the chains converged properly by inspecting caterpillar plots, R-hats and effective sample sizes, and assessed model fit using posterior predictive plots (https://cran.r-project.org/web/packages/bayesplot/vignettes/graphical-ppcs.html). We used the 2.5th and 97.5th percentile from 1000 posterior draws to calculate 95% credible intervals (CrI).

### Accuracy of 3D localization and source-level calculations

We performed two calibration measurements to determine the accuracy of our acoustic tracking system for calculating (i) the 3D-coordinates of bats and (ii) the (apparent) source levels of bat calls. We broadcasted six types of synthetic signals from a custom-built Polaroid speaker placed at 45 different positions relative to the microphone array (3 azimuthal directions×3 elevational directions×5 distances ranging from 3 to 10 m). We analysed the recordings with TOADSuite in the same way as we analysed bat field recordings. We then compared the loudspeaker positions and amplitudes as calculated by TOADSuite to the known spatial positions and previously measured real source levels. The detailed procedures are described in the Supplementary Materials and Methods.

## RESULTS AND DISCUSSION

**Fig. 1.**
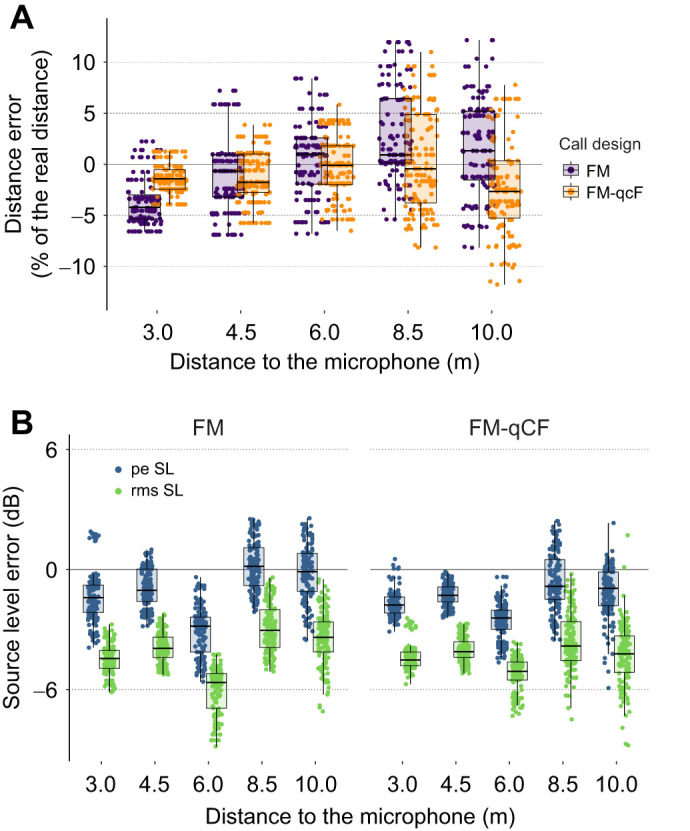
**Accuracy of distance and source-level measurements with a star-shaped four-microphone array and TOADSuite.** Distance (A) and source level (B) error (i.e. difference between real and measured value) as a function of distance to the central microphone, and call shape (FM, frequency modulated; FM-qCF, frequency modulated with a quasi-constant frequency component). Source levels are presented as peak equivalent (pe SL) and root mean square (rms SL) values (B). Raw data (*N*=1374) are shown behind boxplots (median, quartiles and 95th percentiles).

Using a fully calibrated microphone array, we show that wild *P. auritus* flying in an open environment emit echolocation calls with low source level of only 90–99 dB rmsSPL (93–104 dB peSPL) re. 2 µPa at 10 cm distance. Calibrations showed that our system measures amplitude correctly independent of call duration, shape or distance to the array. Our calculated sound source positions showed no systematic error (mean distance error of −0.5% of the real distance, range −9.4–10.9%; Fig. 1) and were not affected by azimuth, elevation, call duration or call shape (Fig. 1; Fig. S1). The localization error increased with increasing loudspeaker distance (Fig. 1), probably due to lower signal-to-noise ratio (SNR). Call frequency and duration were estimated accurately for playback signals having a SNR >20 dB (Fig. S1). Measured source levels were slightly lower than the expected values, with small mean differences for peak values (mean±s.d.: −1.1±1.7 dB) and somewhat larger differences for rms values (–4.2±1.5 dB; Fig. 1). For more details, see Supplementary Materials and Methods.

**Fig. 2.**
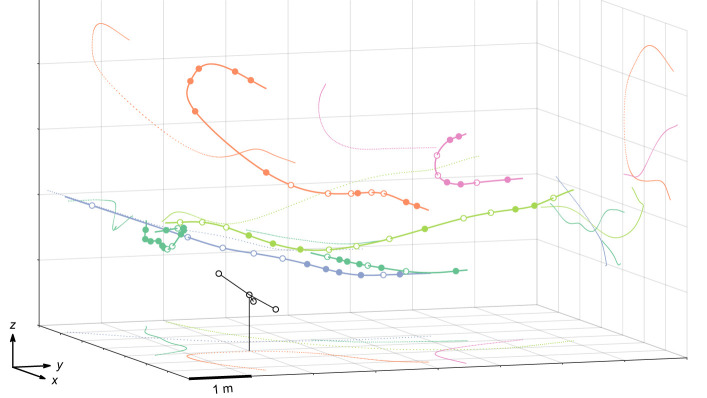
**Three-dimensional trajectories of all five bats whose calls (*N*=98) were analysed.** Filled circles: included calls; open circles: position of calls that were excluded from acoustic analysis. Dotted lines are the projections of the trajectories on the 2D planes. Different colours correspond to different bats (see also Fig. 3). The microphone array is shown in black.

**Fig. 3.**
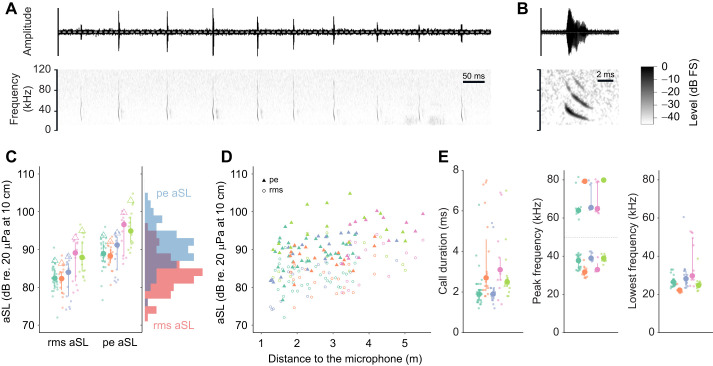
**Spectrogram and call parameters of five brown long-eared bats.** (A,B) Waveforms (top) and spectrograms (bottom) of a 1 s call sequence (A) and a single call (B). FS, full scale. (C) Root mean square (rms) and peak equivalent (pe) apparent source level (aSL) of all analysed calls. Medians (large circles), quartiles (bars) and 90th percentiles (triangles) are shown along with raw data (small circles) per individual bat (colour coded). The histogram on the right shows overall aSL distributions. (D) aSL as a function of the distance to the central microphone. (E) Duration, peak frequency and lowest frequency of all analysed calls. Median and quartiles (large circles and solid lines) and individual calls (small circles) are shown per individual (colour coded). Call duration: the higher values probably include some trailing echoes and/or noise. Peak frequency: medians and quartiles were calculated separately for data below and above 50 kHz, to obtain peak frequencies in the first and second harmonic, respectively (separated by a grey dotted line at 50 kHz). The lack of peak frequencies around 70 kHz in the second harmonic probably originates from a not fully compensated dip in microphone sensitivity around 70 kHz.

We analysed 98 calls of five *P. auritus* flying in a natural open environment (Fig. 2; Fig. S2). We present the median of individual medians along with the range of medians (see Table S1 for individual call data). The calls had a median duration of 2.5 ms (range 1.9–2.8 ms), a median peak frequency of the first harmonic of 37.8 kHz (31.7–39.0 kHz) and a median lowest frequency (−12 dB below peak frequency) of 26.5 kHz (22.2–28.2 kHz; Fig. 3). To estimate real source levels (SL), accounting for the high call directionality and that the call's axis is not necessarily pointing to the microphone, we calculated the 95th percentile of all aSL values per individual. The median (minimum−maximum of individual medians) SL across individuals was 88 dB rmsSPL (86–95 dB rmsSPL) and 94 dB peSPL (92–103 dB peSPL) (Fig. 3C). When adding 4 dB or 1 dB to correct for our system's slight underestimation of the rmsSPL and peSPL, respectively (Fig. 1), *P. auritus* emitted calls with a median source level of 92 dB rmsSPL (90–99 dB rmsSPL) and 95 dB peSPL (93–104 dB rmsSPL). Previous studies estimated 89 and 97 dB peSPL for two individuals flying in a flight room (Waters and Jones, 1995) and 79 dB rmsSPL in a wind tunnel (Jakobsen et al., 2018). Although bats commonly call with lower levels in confined versus in open spaces (Brinkløv et al., 2010; Surlykke and Kalko, 2008), we show that *P. auritus* also emit low-amplitude echolocation calls when flying in open spaces in their natural habitat.

Despite potential methodological challenges, we are confident that we measured typical source levels of *P. auritus* in open space. The apparent increase in source level with distance to the microphone (Fig. 3D) is a recording bias rather than a behavioural response. Intense calls are detected over longer distances than fainter calls. The constant call durations close to the array [mean posterior estimate β=0.057, 95th percentile posterior CrI [−0.091, 0.199], which is an increase of 0.2 ms (10%) over 3 m on average] further confirms that the array did not induce a behavioural artefact. For comparison, common pipistrelles almost double their initial call duration over the same distance (analysis not shown, data from de Framond et al., 2023). While hand-released bats sometimes initially emit atypical echolocation calls (Szewczac, 2004), they soon return to typical echolocation (Obrist and Boesch, 2018) only 5 m post-release (Kohles et al., 2020). We recorded the bats >5 m post-release, so calls should have normalized. Echolocation calls are directional and not always oriented towards the array, which causes underestimated source levels. We thus present the 95th percentile of all echolocation call amplitudes per individual, which is a reliable method to estimate on-axis source levels (Holderied and von Helversen, 2003; Surlykke and Kalko, 2008).

Brown long-eared bat source levels are ∼10 dB lower than those of related low-amplitude species (Corcoran and Conner, 2017; Goerlitz et al., 2010; Lewanzik and Goerlitz, 2018; Seibert et al., 2015). In contrast, the echolocation call levels of aerial-hawking bats in open space are typically 10–100 times (20–40 dB) higher (Boonman et al., 2013; Fujioka et al., 2014; Goerlitz et al., 2020; Holderied and von Helversen, 2003; Holderied et al., 2005; Surlykke and Kalko, 2008). As call level determines the range over which bats can detect obstacles and prey (Goerlitz et al., 2010; Stidsholt et al., 2021), maximizing sensory range probably drove the evolution of high call levels in open-space foragers, up to the physiological limit (Currie et al., 2020). In comparison, the lower source levels of *P. auritus* severely limit their sensory range (Goerlitz et al., 2010), which begs the question of its adaptive value.

One advantage of using low source levels in open environments might unfold in a predator–prey context (Goerlitz et al., 2010). Many moths and other insects possess ears (Kawahara et al., 2019; Ter Hofstede and Ratcliffe, 2016) that enable them to detect bats and initiate evasive responses (Goerlitz et al., 2020; Roeder and Treat, 1957). Low-amplitude ‘stealth’ echolocation enables bats to successfully catch eared prey (Goerlitz et al., 2010; Ter Hofstede and Ratcliffe, 2016) and was thus presented as an adaptation to moth hearing (‘coevolution hypothesis’; Conner and Corcoran, 2012; Fenton and Fullard, 1979; Goerlitz et al., 2010; Surlykke, 1988). In contrast, we argue here that the low source level of *P. auritus* is an adaption to a gleaning foraging strategy (‘habitat adaptation hypothesis’; Lewanzik and Goerlitz, 2018) rather than to moth hearing. Morphological limitations caused by their nasal call emission may also limit a substantial increase of source levels in open environments.

Two lines of evidence support the habitat adaptation hypothesis. First, *P. auritus* are aerodynamically adapted for slow and manoeuvrable flight (Norberg et al., 1987). This is necessary to glean prey in cluttered habitats but disadvantageous when hunting flying prey in open spaces, suggesting that the brown long-eared bat is (still) a primarily gleaning species. For gleaning bats, low-amplitude echolocation prevents the masking of faint prey sounds and prey echoes by intense vegetation echoes (Jacobs and Bastian, 2016). Hence, it seems most plausible that low source levels evolved in a gleaning context as adaptation to cluttered habitats. *Plecotus auritus* may have secondarily exploited their acoustic inconspicuousness for aerial hunting of eared moths, which might explain the substantial proportion of eared moths in their faeces (Andriollo et al., 2019). Such flexible foraging strategies and even foraging niche transitions are common in bats (Arlettaz et al., 2001; Clare et al., 2014; Lewanzik and Goerlitz, 2021; Morales et al., 2019; Ratcliffe and Dawson, 2003).

Second, *P. auritus* emit their calls nasally, in contrast to the oral call emission of high-amplitude aerial-hawking vespertilionid bats. Increasing call source levels is challenging for nasal emitters (Brinkløv et al., 2010, 2011). Only rhinolophid and hipposiderid bats emit high source levels nasally using highly specialized nasal cavities and skulls (Pedersen, 1998; Pedersen and Müller, 2013). Hence, we suggest that *P. auritus* are constrained to low source levels during echolocation. This does not exclude that they could use oral emission to emit loud social calls (as described by Ahlén, 1981). Little is known about these calls, including their source level. The physiological limit to the source levels caused by nasal emission is further supported by one other behaviourally flexible and orally emitting species that dramatically increases source levels (by more than 30 dB) – *Myotis myotis* – and possibly in *O. hemprichii* (no data on emission mode), which increases source level by more than 50 dB when switching from gleaning to aerial hawking (Hackett et al., 2014; Holderied et al., 2011; Stidsholt et al., 2021). Apparently, emitting high source levels during aerial captures is beneficial also for species that otherwise use low source levels when gleaning. Hence, we argue that morphological constraints probably prevent *P. auritus* from emitting high-intensity echolocation calls. Any selection pressure towards high source levels in *P. auritus* has probably been too low because they fly and forage mostly in cluttered environments, and this gives them an advantage in terms of catching eared prey.

In summary, we provide the first data on call source levels in free-flying *P. auritus* and show that – in contrast to some other low-amplitude gleaning species – they do not increase call amplitude when flying in open habitats. Their low call source level is probably an ancestral adaptation to their gleaning strategy. Their nasal call emission probably prevents them from substantially increasing source levels when flying in open environments. We suggest that this putative limitation is beneficial for hawking eared prey in the open air. The morphological limitation scenario contrasts the evolutionary arms-race scenario, in which low-intensity echolocation is a counter-measure to the evolution of ears in prey insects (Goerlitz et al., 2010). Further empirical data on echolocation call parameters, call emission modes, foraging styles and habitat use, as well as ancestral state analyses, are needed to disentangle the evolutionary drivers of extant low-amplitude echolocation.

## Supplementary Material

10.1242/jexbio.245801_sup1 Supplementary information

## ECR Spotlight

This article has an associated ECR Spotlight interview with Léna de Framond.
