# Supplementary material for: Calibrated microphone array recordings reveal that a gleaning bat emits low-intensity echolocation calls even in open-space habitat
Source: J Exp Biol. 2023 Sep 27;226(18):jeb245801. doi: 10.1242/jeb.245801 (PMC10560550; doi:10.1242/jeb.245801)
Supplement: Supplementary information [file jexbio-226-245801-s1.pdf]

Supplementary Materials and Methods

Calibration of the sensitivity of the central microphone

To analyse calls as they were emitted by the bat, call recordings need to be corrected for microphone characteristics. We thus measured the frequency- and direction-dependent sensitivity of the central microphone of the array. We broadcast a calibration sound (1 sec of band-limited white noise (6-95 kHz) and 100-ms pure tones from 6 kHz to 95 kHz in steps of 1 kHz, all corrected for the frequency response of the loudspeaker) from a loudspeaker (custom-built Polaroid speaker). The loudspeaker was placed at 50 cm distance to a ¼-inch measuring microphone with flat frequency response (40BF, with preamplifier 26AC and power module 12AA; GRAS Sound and Vibration, Holte, Denmark) and to the microphone used for recording the bat calls in the field (FG-O, Avisoft Bioacoustics, Glienicke, Germany). The sensitivity of the measuring microphone was calibrated by recording a 1 kHz tone at 94 dB SPL rms (sound calibrator 4231, Brüel & Kjaer, Nærum, Denmark).

Accuracy of 3D-localization and source-level calculations

We performed two calibration measurements to determine the accuracy of our acoustic tracking system (consisting of our array and the TOADSuite software package) for calculating (i) the 3D-coordinates of bats and (ii) the (apparent) source levels of bat calls. As acoustic signals for calibration, we used two types of echolocation-like signals: a linear downward frequency-modulated sweep (FM, 90-25 kHz), and a logarithmic downward frequency-modulated sweep (mimicking a FM-QCF call of e.g., pipistrelle bats; 90-25 kHz). Each signal was generated with three different durations (3, 6, and 9 ms) with a Hann window (to avoid clicks in the loudspeaker) and repeated five times, so that the total playback comprised 30 sweeps, sandwiched between two 100-ms long audible pure tones of 10 kHz (total playback duration 4.8 sec) to indicate the start and end of a playback sequence. The ramp segment of the Hann window is dependent on the total duration of the sound and thus varies with the signal durations. Hence the measured signal duration at -12 dB below peak amplitude will be lower than the specified values.

First, we broadcast this playback from a loudspeaker (custom-built Polaroid speaker) at 3, 4.5, 6, 8.5, and 10 m distance to the microphone array in a large hall. In addition to the on-axis recording (i.e., 0° azimuth and elevation), we also rotated the array relative to the loudspeaker to record the playback from 30° and 60° off-axis in both elevation (below the array's central axis) and azimuth (to the left of the array's central axis). We measured the

Journal of Experimental Biology • Supplementary information

distance between the microphone and the loudspeaker with a laser range-finder (GLM 50C, Bosch, Germany) for every distance and angle adjustment. In total, we thus recorded 1,350 sweeps (30 sweeps per broadcast (2 sweeps × 3 durations × 5 repetitions) × 5 distances × 3 directions in azimuth × 3 directions in elevation).

Second, we analysed the array recordings of the calibration playback with the TOADSuite in the same way as the bat calls recorded in the field (see main text). In short, we band-pass-filtered the recordings (18-90 kHz), automatically detected the broadcast sweeps, determined the time-of-arrival-differences of each sweep between the microphones by cross-correlation, calculated each sweep's three-dimensional position, compensated the sweep's recorded waveform for the frequency- and direction-dependent microphone sensitivity and for the distance-dependent geometric and atmospheric attenuation, and then calculated each sweep's source level from the compensated waveform (both as peak-equivalent SPL and RMS-SPL). This resulted in the 3D-position and source levels of the calibration playback as calculated by the TOADSuite, which we compared in the next steps to the real 3D-positions (as measured by the laser range finder, see above) and the real source level (as calibrated with a measuring microphone, see next step).

Third, we measured the real source level of our playbacks with a calibrated ¼-inch measuring microphone with flat frequency response (40BF, with preamplifier 26AC and power module 12AA; GRAS Sound and Vibration, Holte, Denmark) in an echo-attenuated room. We placed the Polaroid loudspeaker at 50 cm to the measuring microphone and recorded the playback consisting of 30 sweeps. We used custom-written scripts (provided online) in R version 3.6.1 (R Core Team, 2019) to analyse the calibration recording in the same way as the TOADSuite to obtain the real source levels of our playback. Specifically, we band-pass-filtered the recording (18-90 kHz), detected the 30 sweeps, and calculated for each sweep its peak-amplitude from the envelope of the waveform and the RMS-value of the waveform within the -12 dB call duration criterion. By comparing these amplitude measurements to the recording of a calibration tone (94 dB SPL rms, 1 kHz; sound calibrator 4231, Brüel & Kjaer, Nærum, Denmark), we calculated the real source level of both sweeps, both as peak-equivalent value and RMS value, expressed in dB SPL re. 20 µPa at 10 cm distance.

3D Location:

We determined the accuracy of our acoustic tracking system to calculate the three-dimensional coordinates and the source level of a sound source. From the reconstructed 3D-position of the loudspeaker, we calculated the reconstructed distance between loudspeaker

Journal of Experimental Biology • Supplementary information

and central microphone. To quantify the spatial accuracy of our acoustic tracking system, we compared the reconstructed distance to the real distance that we measured with the laser range finder. We express the spatial error between the reconstructed and real distance as the percentage of the absolute difference between the reconstructed and real distance, relative to the real distance:

(1) spatial error (%) = (reconstructed_distance – real_distance) / real_distance * 100.

The effects of the different variables (Distance to the array, Azimuth, Elevation, call duration, call design) on the error in sound source localisation and source level were estimated using a Gaussian Generalized Linear Model (GLM) with identity link function in a Bayesian framework (package *rstanrm* v. 2.21.3, in R v. 4.2.2). Four chains of 5000 iteration each were run (warmup 2500 iteration, thinning rate 1) using the default normal non-informative prior. We examined the quality of model fit following (Gabry, 2022) (package *bayesplot* v. 1.10.1) by examining the MCMC trace, ensuring the Rhats and effective sample size were acceptable, and ensuring that there was minimum autocorrelation within each chain.

The overall mean localization error was close to Zero (-0.5% of the real distance), with a standard-deviation of 4.1% and a range from -9.4 – 10.9% (**Fig. S1**). This small mean error indicates that the tracking system does not systematically over- or underestimate the real position. Calculating a smoothed trajectory of the noisy raw data will thus likely result in a good estimate of the real flight trajectory, and excluding raw data points that are more than ~10% off the smoothed trajectory appears to be justified.

The loudspeaker localization error was independent of the call type (FM or FM-QCF, GLM mean estimate and 95% credible interval: ß (FM-qCF) = -0.91 % [-1.19, -0.63]), and not strongly affected by the azimuth and elevation of the loudspeaker. Generally, the calculated source distance increases with larger elevational off-axis angles (**Fig. S1**, ß(30°) = 2.32% [1.98, 2.66], ß(60°) = 5.36% [5.01, 5.70]) and with smaller azimuthal off-axis-angles (**Fig. S1**, ß(30°) = -1.44% [-1.81, -1.11], ß(60°) = -4.87% [-5.22, -4.51]). The error in localisation caused by distance, elevation and azimuth were all within 6% of the real distance. The distance of the sound source had the most notable effect, causing increasing dispersion of the error, which can likely be attributed to decreasing signal-to-noise ratio (SNR).

In addition to calculating the error for the overall distance, we performed the same calculations separately for each of the three spatial dimensions (X, Y, Z), i.e., we express the spatial error for each dimension by comparing the reconstructed distance to the real distance in that dimension. The X- and Z-axes form the plane of the microphone array, with X being the horizontal and Z the vertical axis, and the Y-axis is the direction away from the array.

Analysing the distance error separately for the three axes showed similar mean errors (±SD) of -1.3±3.9% along the X-coordinate, -1.1±5.8% along the Y-coordinate (distance) and 0.3±5.1% along the Z-coordinate (height). The range of the errors was largest along the distance-axis (Y-coordinate), which is expected for 3D-triangulation methods: X-range = -10.3 – 9.3%, Y-range = -25.5 – 11.2%, Z-range = -13.6 – 10.8%.

Source Level: To quantify the acoustic accuracy of our acoustic tracking system in measuring source levels, we compared the reconstructed source level (SL) to the real source level obtained from the measuring microphone. We express the accuracy of source level reconstruction as the difference between reconstructed and real source level:

(2) SL error (dB) = reconstructed_SL – real_SL.

We used the same statistical method to analyse the effect of call type and distance to the microphone as explained int eh previous section. The mean source levels of our playback signals (relative to 20 µPa and at 10 cm distance to the loudspeaker) were 115.1 dB peSPL (111.8 dB SPL RMS) for the FM signal, and 110.1 dB peSPL (106.1 dB SPL RMS) for FM-QCF signals (±0.1 dB SD in all cases for the 15 sweeps per call type). The source levels calculated by the TOADSuite were slightly lower than these values, resulting in a mean (±SD) source level error of -1.1±1.7 dB for peSPL and -4.2±1.5 dB for rmsSPL (**Fig. S2**). The source level error was independent of the call type (GLM estimates and 95% Credible interval: ß(FM-qCF) = -0.12 dB [-0.25, 0.01]) and the distance to the microphone (ß(distance) = 0.13 dB/m [0.10, 0.15]).

Journal of Experimental Biology • Supplementary information

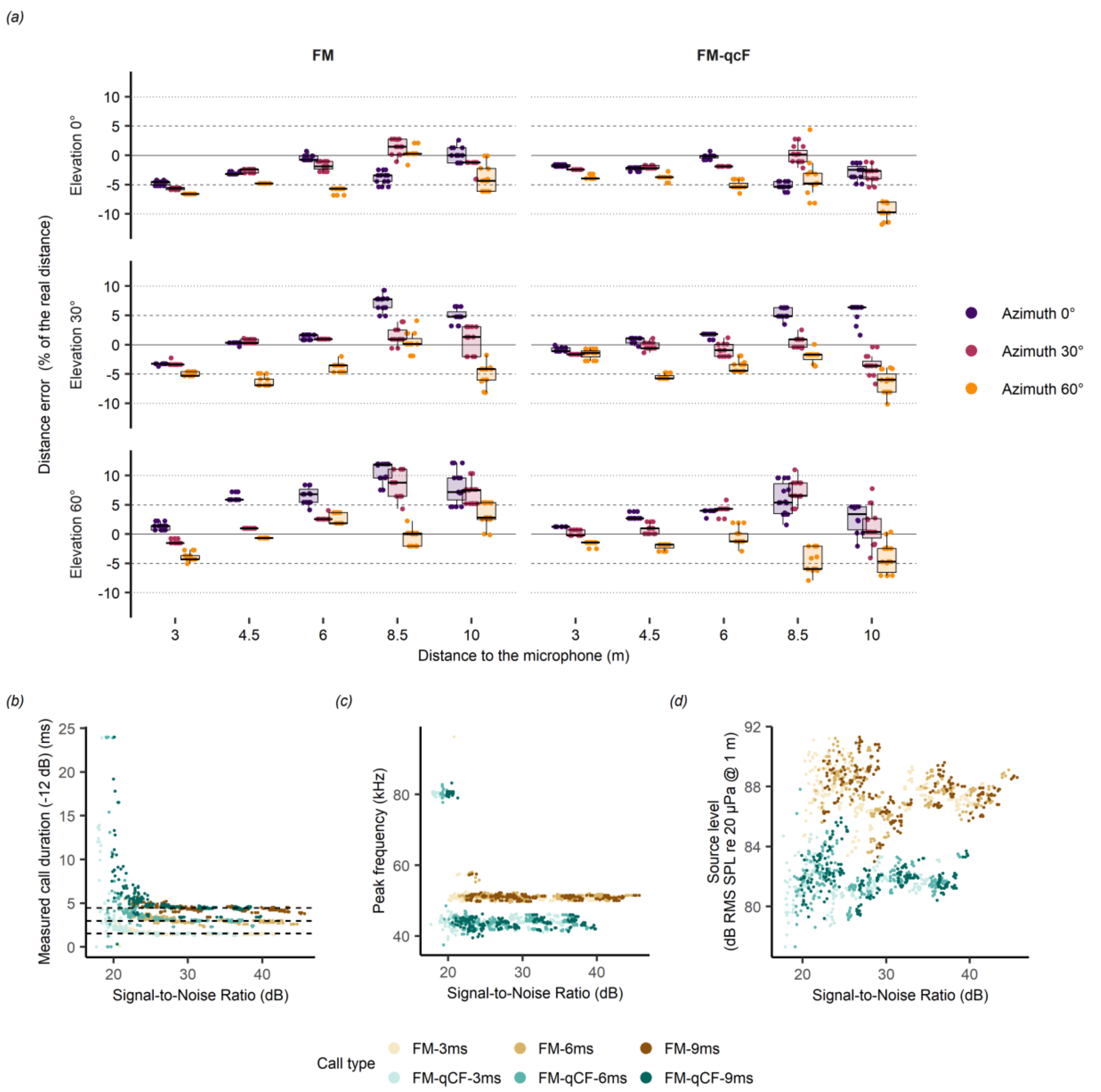

Fig. S1. Accuracy of measuring 3D-positions (A) and call parameters (B-D) with a 60-cm star-shaped microphone array and the TOADSuite

a) The tracking accuracy is indicated by the distance error, which is the difference between the distance calculated with the TOADSuite and the real distance measured with the laser range finder, expressed as a percentage of the real distance. Data is presented as a function of the distance to the microphone, and separated by elevation (panels from top to bottom) and azimuth (color-coded), for two call types (FM signals, left, and FM-QCF signals, right). Raw data is shown behind boxplots, which show median, quartiles, and 95th-percentiles.

b) Measured call duration as a function of the signal-to-noise-ratio. Signal duration and shape are coded with shades and colours. As comparison, the dashed lines show calculated durations for the playback at 1 m distance and recorded with a measurement microphone and analysed independently in R using the same method implemented in the TOADSuite. Note

Journal of Experimental Biology • Supplementary information

that the durations measured with the TOADSuite match the durations on the original playback at high signal to noise levels, but reach unrealistic values when the signal to noise level is below 20 dB.

c) Measured peak frequency as a function of the signal to noise ratio. The peak frequency of the FM-QCF signal is rather constant around 45 kHz because of the long CF component in the call. The FM signals have a peak frequency around 50 kHz because this is the frequency that is most intense after accounting for windowing, and perhaps as a result of the speaker frequency response.

d) Source level calculated with the TOADSuite as a function of the signal to noise ratio.

In summary, source level estimates for peak-values are quite accurate (-1 dB) when averaged over sufficient calls, while the error is larger for average (RMS) estimates (-4 dB). Lower signal-to-noise ratios increased the error but did not lead to any systematic bias in source level measurements (**Fig. S1 D**).

Potential reasons for the larger error in RMS-estimates might be fluctuations in the call envelope caused by interference patterns under real-world recording conditions, incomplete compensation of the microphone's frequency response, and other unknown effects.

Call duration and frequency of calls recorded in the field

The call duration and frequency parameters show some variation and a few outliers (**Fig. 3C**). Analysing call duration as a function of the signal-to-noise ratio (SNR) showed that the call durations and frequencies are reliable for SNR above ~20 dB. For SNR <20 dB, the call duration and frequency of some (but not all) sweeps increased, yet with no obvious pattern regarding which sweep was affected, and no difference between FM and FM-QCF sweeps (**Fig. S1 B,C**). One reason for this pattern might be that with decreasing SNR, the call definition threshold (-12 dB re. maximum amplitude) falls below random fluctuations of the envelope and thus includes noise into the call definition, explaining the few very high call durations in **Fig. 3**.

Overall, we did not find any pattern explaining the extreme outliers in duration and frequency (e.g., long-eared bat calls with long duration were not the same as the calls with very high peak frequency or lowest frequency; no pattern in SNR or localization error) and thus included all data into the analysis.

Journal of Experimental Biology • Supplementary information

Table S1. Individual median (quartiles in brackets below) echolocation call parameters for each bat. N: Number of analysed echolocation calls per bat. aSL: apparent source level (re 20µPa) at 10 cm in front of the bat. We recorded one peak frequency for each call, which sometimes was in the first harmonic, and sometimes in the second harmonic. We grouped calls based on their peak frequency (<50 kHz the measured peak frequency was in the first harmonic, H1, and >50 kHz, the measured peak frequency was in the second harmonic, H2). LoF: lowest frequency.

		Apparent Source level		duration	Peak frequency (kHz)		LoF
**Bat ID**	**N**	**(dB peSPL)**	**(dB SPL rms)**	**(ms)**	**H1**	**H2**	**(kHz)**
**5274**	33	88.9<br>(86.9-91.6)	82.4<br>(80.1-85.1)	1.9<br>(1.6-2.2)	37.8<br>(33.6-40.4)	64.0<br>(62.6-68.9)	26.5<br>(24.7-27.8)
**5280**	19	88.3<br>(86.2-90.4)	82.3<br>(78.9-84.0)	2.7<br>(2.1-4.9)	31.8<br>(29.5-33.3)	79.3<br>(78.8-79.8)	22.3<br>(21.8-22.7)
**5295**	17	91.2<br>(84.2-93.4)	84.0<br>(77.3-86.4)	1.9<br>(1.7-2.2)	39.0<br>(37.9-42.1)	65.5<br>(64.9-77.7)	28.3<br>(25.3-32.3)
**5297**	13	96.6<br>(88.2-97.9)	89.1<br>(81.4-92.0)	3.1<br>(2.5-3.9)	33.0<br>(32.3-37.9)	64.9<br>(63.5-79.0)	29.8<br>(27.8-50.2)
**5298**	16	94.9<br>(91.7-99.0)	87.9<br>(84.2-92.6)	2.5<br>(2.3-2.8)	39.0<br>(37.8-39.8)	79.9<br>(79.5-80.3)	24.8<br>(23.9-26.1)

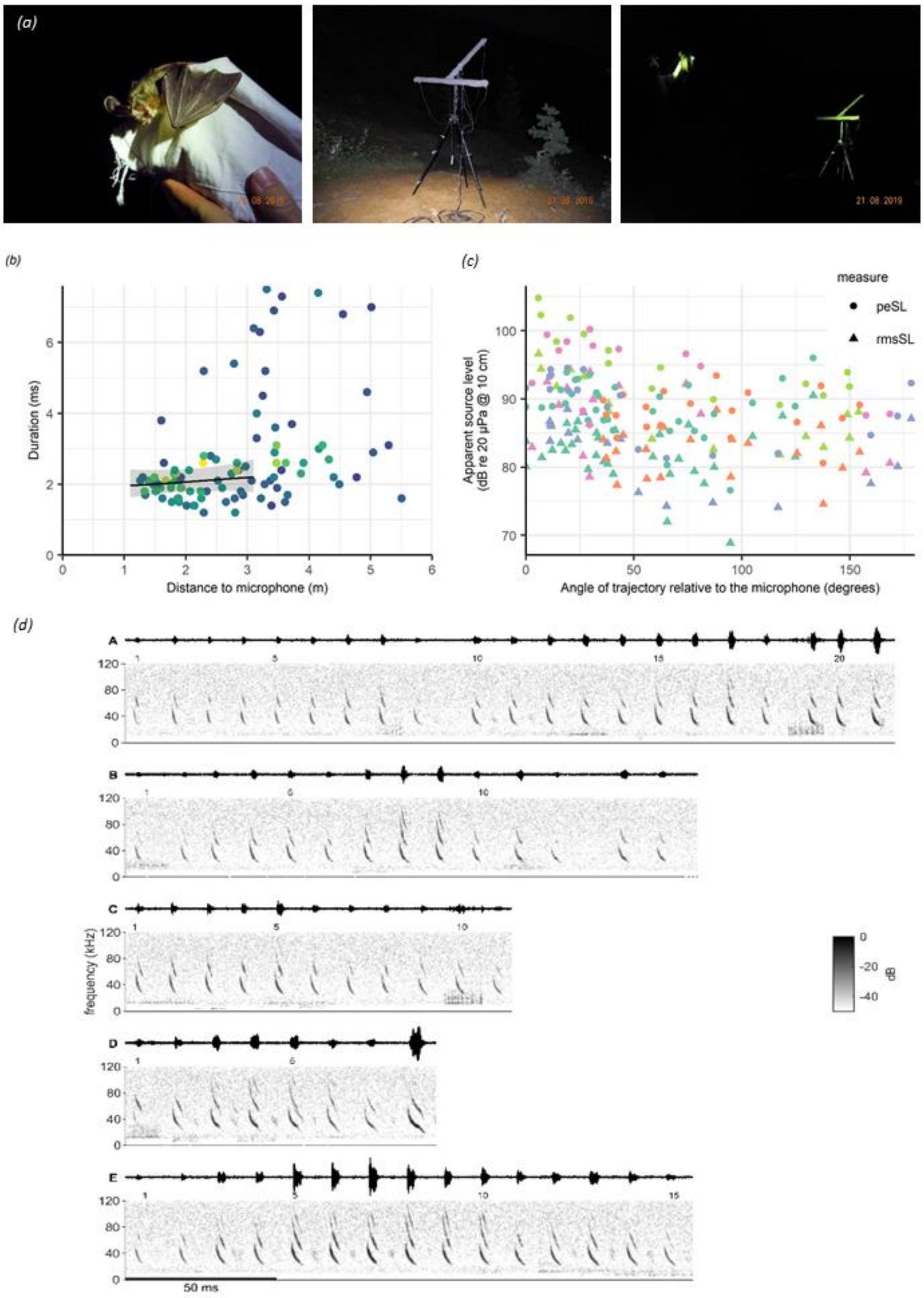

Fig. S2. Experimental situation, effect of flight behaviour on call parameters and recorded calls.

a) From left to right, one individual of a brown-long-eared bat, *Plecotus auritus*, before release, the microphone array in the field (note the open surroundings), and hand release of one individual (left) in front of the microphone array (right).

b) Effect of the distance between the bat and the microphone array on call duration. Raw data points are color-coded according to the signal to noise ratio. Note that many points

have a very low signal to noise ratio (<20 dB) at distances >3 m from the microphone array, which are also the calls with high call duration and frequency. Within 3 m of the microphone array (where the signal to noise ratio is high for most calls), the call duration did not change with distance to the array (Linear Mixed Effect Model of the log (call duration) as a function of distance to the array, bat identity as a random effect, ß(distance) = 0.06, 95% credible interval [-0.09, 0.20], that is an increase by .2 ms on average over 3 m).

c) Source level as a function of the angle between direction from the bat to the microphone array and the bat's actual flight direction, color-coded per individual.

d) Waveforms (top) and spectrograms (bottom) of the analysed calls of the five bats. Note that for better visibility, only the concatenated individual detected and analysed calls are shown, not the complete recorded call sequences including silent phases between calls. The letters refer to bat identity in Figure 2 in the main manuscript.

Supplementary References

Gabry, J. (2022). Graphical posterior predictive checks using the bayesplot package.

Gabry, J. and Mahr, T. (2022). bayesplot: Plotting for Bayesian Models.

Goodrich, B., Gabry, J., Ali, I. and Brilleman, S. (2020). rstanarm: Bayesian applied regression modeling via Stan.

R Core Team (2019). R: A language and environment for statistical computing.

Journal of Experimental Biology • Supplementary information
